# Testing a non-destructive assay to track *Plasmodium* sporozoites in mosquitoes over time

**DOI:** 10.1186/s13071-023-06015-5

**Published:** 2023-11-04

**Authors:** Catherine E. Oke, Sarah E. Reece, Petra Schneider

**Affiliations:** 1 Institute of Ecology and Evolution, School of Biological Sciences, University of Edinburgh, Edinburgh, UK; 2 Institute of Immunology and Infection Research, School of Biological Sciences, University of Edinburgh, Edinburgh, UK

**Keywords:** Extrinsic incubation period, *Anopheles stephensi*, *Plasmodium berghei*, *Plasmodium chabaudi*, Malaria transmission

## Abstract

**Background:**

The extrinsic incubation period (EIP), defined as the time it takes for malaria parasites in a mosquito to become infectious to a vertebrate host, is one of the most influential parameters for malaria transmission but remains poorly understood. The EIP is usually estimated by quantifying salivary gland sporozoites in subsets of mosquitoes, which requires terminal sampling. However, assays that allow repeated sampling of individual mosquitoes over time could provide better resolution of the EIP.

**Methods:**

We tested a non-destructive assay to quantify sporozoites of two rodent malaria species, *Plasmodium chabaudi* and *Plasmodium berghei*, expelled throughout 24-h windows, from sugar-soaked feeding substrates using quantitative-PCR.

**Results:**

The assay is able to quantify sporozoites from sugar-soaked feeding substrates, but the prevalence of parasite-positive substrates was low. Various methods were attempted to increase the detection of expelled parasites (e.g. running additional technical replicates; using groups rather than individual mosquitoes), but these did not increase the detection rate, suggesting that expulsion of sporozoites is variable and infrequent.

**Conclusions:**

We reveal successful detection of expelled sporozoites from sugar-soaked feeding substrates. However, investigations of the biological causes underlying the low detection rate of sporozoites (e.g. mosquito feeding behaviour, frequency of sporozoite expulsion or sporozoite clumping) are needed to maximise the utility of using non-destructive assays to quantify sporozoite dynamics. Increasing detection rates will facilitate the detailed investigation on infection dynamics within mosquitoes, which is necessary to explain the highly variable EIP of *Plasmodium* and to improve understanding of malaria transmission dynamics.

**Graphical Abstract:**

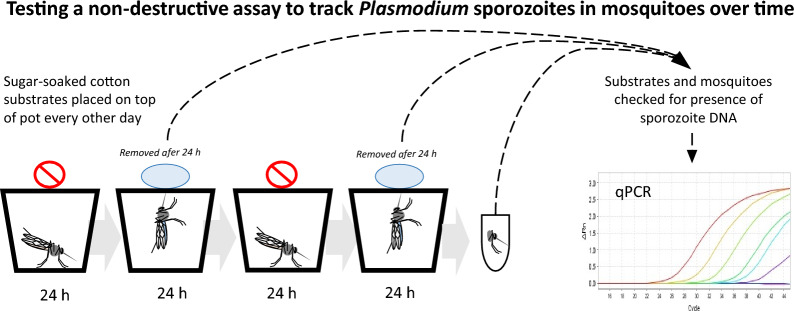

## Background

Malaria, caused by *Plasmodium* parasites, [1] is transmitted between vertebrate hosts by anopheline mosquito vectors. Within the vector, parasites must mate, reproduce, traverse the midgut wall, replicate extensively and then migrate to the salivary glands. Only after all these processes (defined as sporogony) are completed can parasites infect a new vertebrate host. The time it takes for parasites to complete their development in the vector (the extrinsic incubation period [EIP] [2]) is usually reported to be 10–20 days [3, 4]. This is surprisingly long given that only a very small proportion of mosquitoes live longer than 3 weeks in the field [5–7].

Small changes in the EIP can have a large effect on the number of mosquitoes living long enough to become infectious, making it a crucial parameter for transmission potential (i.e. *R*_0_) [3]. Although the historical assumption that the EIP only depends on temperature [8–10] has been overturned [3], current understanding of other sources of variation in EIP remains limited. Variation in the EIP is associated with environmental factors, such as temperature and availability of the vector’s resources, along with intrinsic differences between *Plasmodium* species. For example, *Plasmodium mexicanum*, vectored by the short-lived sand fly, has a shorter EIP [2], whereas *Plasmodium berghei* has a longer EIP, partly due to adaptation to the lower temperature of their vector’s habitat [11]. In comparison, *Plasmodium chabaudi* and *Plasmodium falciparum* have similar development times, with the development time of the latter speeding up when mosquitoes receive an additional blood meal [12]. Furthermore, longer EIPs have been observed in *P. falciparum*-infected mosquitoes with lower salivary gland burdens [13]. Why malaria parasites cannot develop faster is a longstanding mystery and highlights the need to investigate whether constraints (such as the dynamics of resource availability within mosquitoes) and/or benefits to the parasite (such as transmission correlating positively with sporozoite number) shape the EIP [6].

Explaining the EIP is challenging because it is most commonly approximated as the time at which sporozoites are first visible in the salivary glands [3]. However, sporozoites may require a period of maturation to become infectious; heterogeneous gene expression suggests that not all sporozoites residing in the salivary glands are infectious [14]. Additionally, salivary gland sporozoites may need to exceed a density threshold for onwards transmission to be likely. Transmission probability significantly increases above 10,000 sporozoites for *Plasmodium yoelii* [15], even though only tens to low hundreds of sporozoites are thought to be expelled during transmission [13, 16–18], but studies using *P. falciparum* suggest a lower (> 1000) threshold [19]. Furthermore, some infected mosquitoes do not expel any sporozoites [13, 20, 21], further complicating the correlation between salivary gland sporozoites and transmission probability.

Tools for estimating the EIP are also problematic; the EIP is typically estimated from terminal (i.e. destructive) sampling of a subset of mosquitoes from the population at intervals during sporogony. Sporozoites are usually assayed following dissection of the salivary glands for microscopic detection [22], or by molecular assays from (bisected) mosquitoes [13, 23]. These methods have several limitations. First, terminal sampling prevents tracking individual mosquitoes over time, so EIP is estimated at the population level. However, while population-level measures such as the median EIP (EIP_50_) are useful for modelling purposes [3], they do not consider the individual variation important for linking vector-parasite-environment interactions with the EIP and infectiousness [24, 25]. Second, processing a subset of mosquitoes every few days is laborious and requires large numbers of infected mosquitoes.

Sporozoites are expelled during sugar feeding [26, 27], and expelled sporozoites have a greater chance of being infectious than those in the glands. Thus, using a non-destructive assay that quantifies expelled sporozoites on sugar-soaked feeding substrates allows the infections of individual mosquitoes to be followed over time and can improve resolution of the EIP. Non-destructive sugar-based assays to quantify sporozoites, using PCR or immunoblotting detection of circumsporozoite protein, have been tested for groups or single mosquitoes infected with *P. falciparum* [24, 26–29] and for groups of *P. berghei*-infected mosquitoes [28, 30], with some success. While assays able to detect sporozoites from groups of mosquitoes are useful for field surveillance of malaria prevalence [26–28], assays sensitive enough to detect sporozoites from individual mosquitoes provide the best resolution of EIP and its determinants. Furthermore, investigating the ecological and evolutionary determinants of the EIP, including the impact of host factors, requires model systems in which the full life-cycle can be manipulated in vivo. Due to their tractability, rodent malarias are ideal, but there is no assay available for individual mosquitoes infected with these *Plasmodium* species. The most used model, *P. berghei*, is useful for proof of principle investigations of EIP-related questions, including onward transmission to a vertebrate host, but *P. chabaudi* provides a unique opportunity to investigate EIP at a similar parasite density and temperature [31] to *P. falciparum*.

Here, we tested a non-destructive method to detect *P. berghei* and *P. chabaudi* sporozoites from mosquitoes’ sugar-soaked feeding substrates. We compared how well this technique performs for *P. berghei* and *P. chabaudi*, which have different optimal temperatures for sporogony and therefore different EIPs. We demonstrated that *Plasmodium* DNA from both species can be detected and quantified from sugar-soaked feeding substrates. However, while the detection rate for sporozoites in mosquito expectorates in our study is similar to that reported in other studies [24, 28], parasite prevalence was low. We discuss potential explanations for low parasite prevalence in individual mosquito’s expectorate and suggest further improvements to mosquito sugar feeding assays.

## Methods

The quantitative-PCR (qPCR) assay to quantify *Plasmodium* sporozoites was validated and used to determine the best sugar-soaked feeding substrate for the assay, the range of sporozoite DNA concentrations that can be recovered from the substrates, as well as optimal storage conditions and sugar concentrations to minimise DNA degradation. Subsequently, the recovery of expelled sporozoites from individual mosquitoes was investigated, as well as methods to increase the detection of expelled parasites.

### Mosquitoes and malaria infections

*Anopheles stephensi* SD500 mosquitoes were reared at 26 °C and 70% relative humidity under a 12:12-h (light:dark) photoperiod, with ad libitum access to 8% fructose solution post-emergence. Transmission to mosquitoes was achieved by allowing the mosquitoes to blood-feed on mice (8-to-10-week-old male C57Bl/6 mice) with microscopy-confirmed gametocytes of either *P. berghei* ANKA or *P. chabaudi* genotype ER (following [31, 32]). Mosquitoes used for *P. berghei* transmissions were gradually acclimatised to 21 °C prior to infectious blood meals. All mosquitoes were starved for 24 h before infection, and unfed females were removed on day 1 post-infectious blood meal (pIBM).

### DNA extraction

DNA from microscopy-quantified blood-stage parasites [33] was extracted from 5 µl of blood using a semi-automatic Kingfisher Flex Magnetic Particle Processor and MagMAX™-96 DNA Multi-Sample Kit (Thermo Fisher Scientific, Waltham, MA, USA) as per [34], and was frozen at − 20 °C until use. These blood-stage DNA samples were used to determine qPCR efficiency and the limit of detection (LOD).

DNA was extracted from head/thorax mosquito samples and feeding substrates following the CTAB-based phenol–chloroform extraction method of Chen et al. [35] with minor modifications (as described in Schneider et al. [33]). Extracted DNA was eluted in 30 µl (mosquitoes, supplemented feeding substrates) or 16 µl (mosquito expectorate substrates) of water and frozen at − 20 °C until use. DNA extracts from mosquitoes, but not from feeding substrates, were diluted fourfold to reduce the effect of inhibitors originating from mosquito material on the performance of the PCR. All PCR reactions were run using 7 µl of (diluted) DNA extracts, and data are presented as genomes/PCR, unless stated otherwise, to account for differences in sample processing.

### Quantification of *Plasmodium* by qPCR

Both *P. berghei* and *P. chabaudi* were assayed by a qPCR targeting a region of the* 18S* ribosomal RNA (rRNA) gene that is highly conserved among *Plasmodium* species [36]. Parasite genomes were quantified by comparing the threshold cycle (Ct) against a standard curve, generated from DNA extracted from blood-stage parasites of either *P. berghei* ANKA or *P. chabaudi* genotype ER (see section DNA extraction). Due to differences in the efficiency of extractions between blood and mosquito/substrate, absolute numbers of sporozoites may be slightly overestimated, but relative differences between experimental groups remain the same. Negative water controls were included to identify false positives.

### Optimising the assay

The assay was optimised using two reference DNA samples from sporozoite-infected mosquitoes. DNA samples from *Plasmodium-*infected mosquitoes, shown by qPCR to have high sporozoite loads, were pooled to create one reference DNA sample for *P. berghei* (2967 genomes/µl) and one for *P. chabaudi* (5099 genomes/µl). These reference samples were used to determine: (i) which type of feeding substrate type returned an optimal DNA yield; (ii) whether DNA could be detected across a range of concentrations; (iii) whether DNA degradation occurred during the collection period; and (iv) whether sugar content impacted DNA yield.

The most suitable feeding substrate was selected by comparing the recovery of parasite DNA from 15 mg cotton wool, a 1-cm^2^ cotton pad (Boots UK Ltd., Nottingham, UK) or a 1-cm^2^ filter paper (Whatman No. 1; Whatman plc, Maidstone, UK). Each substrate (*n* = 3 per substrate type) was soaked in 8% fructose, supplemented with 5 µl of *P. berghei* or *P. chabaudi* reference DNA and stored at 26 °C and 70% relative humidity for 24 h to mimic housing conditions for *P. chabaudi*-infected mosquitoes. Reduced DNA yields are expected at higher temperatures [37], so these conditions were assumed to provide a conservative estimate of assay performance for *P. berghei*. DNA yield was calculated by comparing qPCR results directly from reference DNA with those from spiked feeding substrates, accounting for any dilutions during sample processing. Subsequent tests were conducted using cotton wool, and all substrates were kept under the same conditions as described above. Second, to confirm that DNA could be consistently detected and quantified across a range of concentrations, cotton wool substrates were supplemented with 5 µl of serial dilutions of *P. berghei* (neat 10^0^ to 5 × 10^–3^ dilution) or *P. chabaudi* (neat 10^0^ to 5 × 10^–4^ dilution) reference samples, (*n* = 3 per dilution/species), immediately after soaking in 8% fructose (time point 0 h). Linearity of quantification and the limit of quantification (LOQ), relative to the LOD, was quantified. Third, DNA degradation under conditions mimicking mosquito housing was tested by comparing DNA recovery from cotton wool supplemented with 5 µl reference sample (10^0^ to 10^–2^ dilution for each species), either immediately after soaking in 8% fructose (time point 0 h) or at collection (time point 24 h) (*n* = 3 per time point/species). Finally, the impact of sugar concentration on DNA yield was tested by soaking cotton wool substrates in distilled water, 1% or 8% fructose supplemented with 5 µl of serial dilutions of *P. berghei* or *P. chabaudi* reference samples (neat 10^0^ to 10^–2^ dilution, *n* = 3 per dilution/species). Parasite DNA recovery was compared between the three sugar concentrations.

### Testing the assay on mosquito expectorate samples

**Fig. 1:**
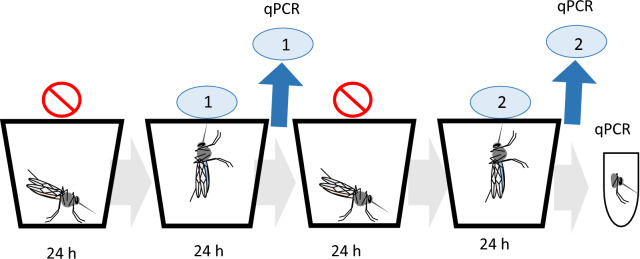
Timing of cotton substrate collection from *Plasmodium*-infected mosquitoes. Mosquitoes were moved to paper cups and starved for 24 h, then provided access to a sugar-soaked feeding substrate. After 24 h of feeding, the substrate was collected for sporozoite detection by qPCR. This cycle was repeated twice, such that two substrates were collected per mosquito. The cycle started on day 22 pIBM for *P. berghei* and day 12 pIBM for *P. chabaudi*.* pIBM* post-infectious blood meal, qPCR quantitative-PCR

To collect expectorate samples, mosquitoes were moved to paper cups, either individually (*P. berghei* *n* = 13; *P. chabaudi* *n* = 10) or in groups (*P. berghei*, 4 mosquitoes/cup, *n* = 5 cups). To increase the likelihood of mosquitoes feeding on the sugar-soaked substrate, mosquitoes were starved for 24 h prior to being provided with the feeding substrate, which was collected 24 h later and stored at − 20 °C until DNA extraction. This 2-day starvation–feeding cycle was repeated twice during days 22–25 pIBM for *P. berghei* and during days 12–15 pIBM for *P. chabaudi* (Fig. 1). After both sets of expectorate samples were collected, mosquitoes were anaesthetised on ice and bisected following [23]. Head-thorax specimens were stored at − 20 °C until DNA extraction and subsequent salivary gland sporozoite quantification by qPCR. Only data from sporozoite-infected mosquitoes that survived for the entire experiment were included in all analyses (1 *P. berghei* and 1 *P. chabaudi* uninfected individual were excluded; no uninfected mosquito groups were detected).

### Statistical analysis

Data analyses were performed using R v. 4.1.3 ® Foundation for Statistical Computing, Vienna, Austria). Linear models were used to determine PCR efficiency and compare the efficiency between species. The absolute LOD [38], defined as the minimum concentration that can be detected with a sensitivity of 100%, was determined using plateau-linear models fitted to qPCR Ct values and associated genome counts (SSplin, *nlraa* package [39]). These models predict the switching point from a plateau to a linear slope, thus indicating when the qPCR true positivity rate dropped to < 1. Parasite densities below the LOD were set to zero. To determine the most suitable substrate and sugar concentration, and to test for DNA degradation over time, linear models were used to investigate the effect of the variable tested (substrate, sugar or time), parasite species, DNA concentration (if relevant) and all their interactions on Ct value. DNA yield across *Plasmodium* concentrations was analysed using linear models for *P. chabaudi* and the SSplin function for *P. berghei*, for which this non-linear model fitted better than a linear regression (change in corrected Akaike information criterion [ΔAICc] > 2).

The presence/absence of parasite DNA from expelled sporozoites over time was tested using binomial generalised linear models (glm), including an interaction between *Plasmodium* species and salivary gland burden. Further binomial glms were used to test whether processing a larger proportion of the mosquito expectorate DNA extract (summing parasite densities detected in two qPCR replicates) or collecting expectorates from small groups of *P. berghei-*infected mosquitoes rather than from individuals improved detection rates, including species and either replicate or grouping, as well as their interaction, in the models. Negative binomial models (glm.nb function, *MASS* package [40]) were used to investigate whether the number of expelled sporozoites on positive substrates was affected by: (i) day and salivary gland burden, and how this varied by species; (ii) summing parasite densities from two qPCR replicates, by species; (iii) grouping *P. berghei*-infected mosquitoes, by day; and (iv) whether salivary gland sporozoite burden differed between species.

Models were minimised using likelihood ratio tests, and AICc for non-nested models. All models met model assumptions, confirmed by simulating and plotting residuals using the *DHARMa* package [41]. Confidence intervals (CIs) were obtained from statistical models or, in the case of confidence intervals for quotients, using Fieller’s method [42].

## Results

### Validation of qPCR for sporozoite detection: true and false positivity

**Fig. 2:**
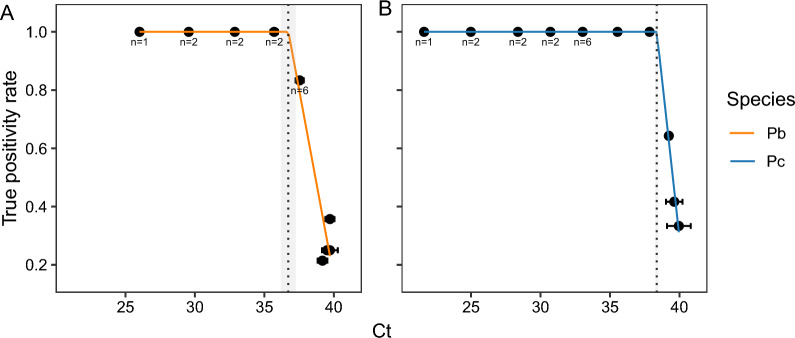
True positivity rates, determined from quantification of a serial dilution of DNA from blood-stage parasites, for *Plasmodium berghei* (**a**, orange) and *Plasmodium chabaudi* (**b**, blue). Mean Ct values ± SEM are presented for *P. berghei* (0.001–6428 genomes/PCR) and *P. chabaudi* (0.05–75,461 genomes/PCR), tested in *n* = 12 replicates unless stated otherwise on the graph. The LOD (concentration at which the true positivity rate drops below 1) ± SEM, predicted using a plateau-linear function is 4.4 (Ct 36.7 ± 0.5) or 0.8 (Ct 38.4 ± 0.1) genomes/PCR for *P. berghei* and *P. chabaudi*, respectively (dotted lines ± shading). Ct, Cycle threshold; LOD, limit of detection; Pb, *Plasmodium berghei*; Pc, *Plasmodium chabaudi*; SEM, standard error of the mean

The qPCR assay targeting the* 18S* rRNA gene has been previously validated for sporozoite detection, achieving a 95% amplification efficiency and a LOD < 10 parasites/PCR [36]. We replicated this high qPCR performance using DNA extracted from blood stages of *P. berghei* (0.5–6428 genomes/PCR) or *P. chabaudi* (0.2–75,461 genomes/PCR reaction), achieving an amplification efficiency of 99.5 ± 2.6% (*R*^2^ = 0.99), with equal performance between species (log_10_ parasite density by species interaction: *F*_(1,20)_ = 0.51, *P* = 0.48). Although quantification is accurate when low parasite densities are detected, detection rates drop at lower densities. The LOD (the concentration at which the true positivity rate drops below 1) was 4.4 genomes/PCR (mean Ct ± standard error of the mean [SEM]: 36.7 ± 0.5) for *P. berghei* (Fig. 2a) and 0.8 genomes/PCR reaction (mean Ct ± SEM: 38.4 ± 0.1) for *P. chabaudi* (Fig. 2b). At parasite densities below the LOD (i.e. higher Ct values), the rate of false negatives increases and these densities were set to zero. False positives (water samples) were not detected at concentrations above the LOD for either* Plasmodium* species.

### Optimising the assay

**Fig. 3:**
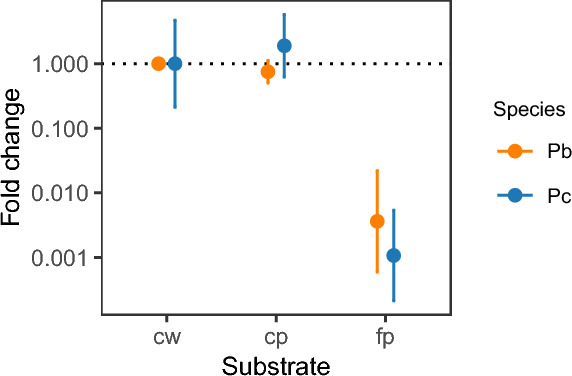
Relative DNA yield from substrates soaked in 8% fructose and supplemented with reference DNA extracted from *P. berghei*-infected (orange) or *P. chabaudi*-infected (blue) mosquitoes. DNA yield is presented as the mean fold difference ± 95% CI (*n* = 3/substrates/species) relative to the mean DNA yield for cotton wool for each species and displayed on a log_10_ scale to clearly visualise both increased and decreased DNA yield. CI, Confidence interval; cp, cotton pads; cw, cotton wool; fp, filter paper; Pb, *Plasmodium berghei*; Pc, *Plasmodium chabaudi*

To determine the most suitable feeding substrate for the assay, we tested three different substrates, all soaked in 8% fructose and supplemented with 5 µl reference DNA from *P. berghei*- or *P. chabaudi*-infected mosquitoes: filter paper, cotton wool and cotton pads. DNA yield varied by substrate type and parasite species (substrate × species interaction: *F*_(2,12)_ = 4.42, *P* = 0.036). Compared to cotton wool extraction efficiencies (21% for *P. chabaudi*, 25% for *P. berghei*), cotton pads resulted in similar DNA yields, while filter paper yielded 933-fold (95% CI: 186–4683) and 276-fold (95% CI: 45–1675) lower DNA for *P. chabaudi* and *P. berghei*, respectively (Fig. 3). Based on DNA yield and ease of use, we selected cotton wool as the feeding substrate for the remainder of this study.

**Fig. 4:**
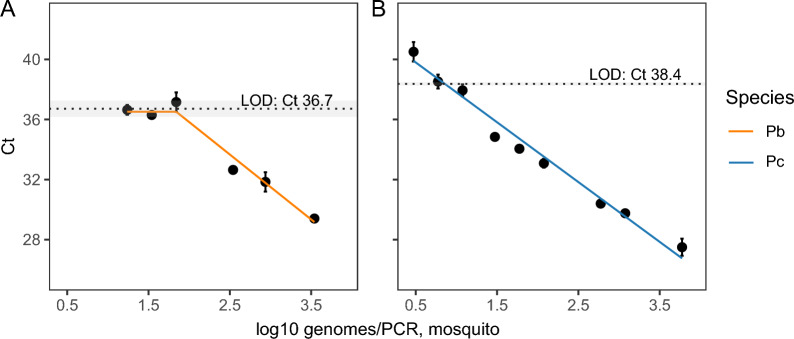
Linearity of quantification for *P. berghei* (orange; **a**) and *P. chabaudi* (blue; **b**) reference DNA (cotton wool, 8% fructose). Mean Ct values ± SEM are presented for the reference DNA originating from infected mosquitoes (log_10_ genomes/PCR, mosquito), ranging from 17 to 3461 genomes/PCR (*P. berghei*) or from 3 to 5949 genomes/PCR (*P. chabaudi*) (*n* = 3/concentration/species). The LOQ (Ct at which samples can be detected but quantification becomes inaccurate) is determined as the switch point where the plateau ends. The dashed line shows the Ct value associated with the LOD ± SEM (see Fig. 2) for blood samples. Ct, Cycle threshold; LOD limit of detection; LOQ, limit of quantification; Pb, *Plasmodium berghei*; Pc, *Plasmodium chabaudi*; SEM, standard error of the mean

**Fig. 5:**
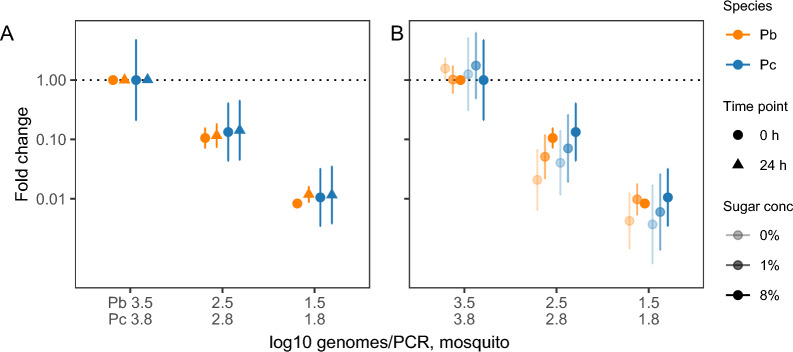
Relative DNA yield in response to storage time (**a**) and sugar concentration (**b**) for reference DNA quantified from cotton wool substrates. Data points present mean fold change ± 95% CI, relative to substrates supplemented with neat reference DNA at time point 0 (**a**) or soaked in 8% fructose (**b**), for each species. Dilutions of reference DNA, originating from infected mosquitoes (log_10_ genomes/PCR, mosquito) range from 35 to 3461 genomes/PCR (*P. berghei*, orange) or from 59 to 5949 genomes/PCR (*P. chabaudi*, blue) (*n* = 3/concentration/group). CI, Confidence interval; Pb, *Plasmodium berghei*; Pc, *Plasmodium chabaudi*

Assay performance for cotton wool was tested by supplementing cotton wool substrates with 5 µl of a serial dilution of reference DNA for *P. berghei* (17–3461 genomes/PCR) or *P. chabaudi* (3–5949 genomes/PCR) (Fig. 4). Non-linearity for *P. berghei* samples with Ct > 36.5 ± 0.4 showed that quantification became inaccurate at parasite densities < 50 genomes/PCR. This switching point is referred to as the LOQ and occurred at a similar Ct value as for *P. berghei* blood samples (36.7 ± 0.5; dotted line; Fig. 4a). For *P. chabaudi*, the linear dynamic range covered all tested parasite densities, suggesting that we can confidently detect and quantify *P. chabaudi* genomes from cotton wool substrates up to the LOD as determined by the *P. chabaudi* blood samples above (Ct 38.4 ± 0.1; Fig. 4b). The slopes in Fig. 4 were steeper than expected, indicating PCR efficiencies of 70.4 ± 8.9% for *P. berghei* and 78.2 ± 4.5% for *P. chabaudi*, which could be explained by covering a wider range of DNA concentrations: DNA quantities were underestimated for low-density samples, with Ct values at/above the LOD. To maximise the chances of mosquitoes feeding on the substrate and expelling sporozoites, access to substrates lasted for 24 h. Because the conditions in which mosquitoes were kept may not have been optimal for the preservation of DNA, we investigated DNA degradation over 24 h. Specifically, we compared DNA yield from cotton wool supplemented with DNA at the time of sugar soaking (time point 0) or supplemented with DNA at the time of collection 24 h later (time point 24). As expected, lower DNA concentrations resulted in a lower DNA yield (DNA: *F*_(1,33)_ = 2036.6, *P* < 0.001). While the absolute number of genomes varied between species, reflecting the higher parasite densities in the *P. chabaudi* compared to the *P. berghei* reference sample (species: *F*_(1,33)_ = 310.9, *P* < 0.001), quantification was equally efficient in both species (DNA × species interaction: *F*_(1,31)_ = 0.04, *P* = 0.87). We did not observe DNA degradation after 24 h of storage for either species (time × species interaction: *F*_(1,29)_ = 0.0004, *P* = 0.98; time: *F*_(1,32)_ = 3.71, *P* = 0.063), across all parasite densities (time × DNA × species interaction: *F*_(1,29)_ = 1.76, *P* = 0.20; time × DNA interaction: *F*_(1,31)_ = 0.11, *P* = 0.75) (Fig. 5a).

Mosquito feeding substrates have a high sugar concentration (usually fructose or glucose), which may affect the extraction efficiency and subsequent DNA amplification. To test whether the sugar content of the substrate affected DNA yield, we compared DNA recovery from cotton wool substrates soaked in 0, 1 or 8% (w/v) fructose, and supplemented with reference DNA at time point 0. Our analysis confirmed higher parasite densities in the *P. chabaudi* reference sample, compared to *P. berghei* reference sample (species: *F*_(1,49)_ = 80.4, *P* < 0.001), that lower DNA concentrations result in lower DNA yields (DNA: *F*_(1,49)_ = 639.0, *P* < 0.001) and that quantification of DNA is equally efficient for both species (DNA × species interaction: *F*_(1,48)_ = 0.84, *P* = 0.36). Sugar concentration impacts DNA yield (sugar: *F*_(2,49)_ = 8.10, *P* < 0.001); lower concentrations reduce the yield compared to 8% fructose by 1.5-fold (95% CI: 1.1–2.1) for 1%, and 2.4-fold (95% CI: 1.7–3.3) for 0% (Fig. 5b), in the same manner across parasite species and densities (sugar × DNA ×species interaction: *F*_(2,42)_ = 0.56, *P* = 0.58; sugar × species interaction: *F*_(2,44)_ = 0.13, *P* = 0.88; sugar × DNA interaction: *F*_(2,46)_ = 1.30, *P* = 0.28). Together, these results confirm that collecting mosquito expectorate over a period of 24 h on substrates soaked in 8% fructose are the optimal assay conditions.

### Testing the assay using mosquito expectorate samples

Following optimisation of the assay using reference DNA, we tested the assay’s performance using mosquito expectorate samples. We allowed *Plasmodium-*infected mosquitoes, housed individually (*n* = 12 *P. berghei*, *n* = 9 *P. chabaudi*) or in small groups (*n* = 5 groups of 4 mosquitoes/group for *P. berghei*), to feed for 24 h on cotton wool substrates soaked in 8% fructose, which were collected twice per (group of) mosquito(es). We then compared the prevalence and density of parasite DNA in the feeding substrates between species and by group size.

**Fig. 6:**
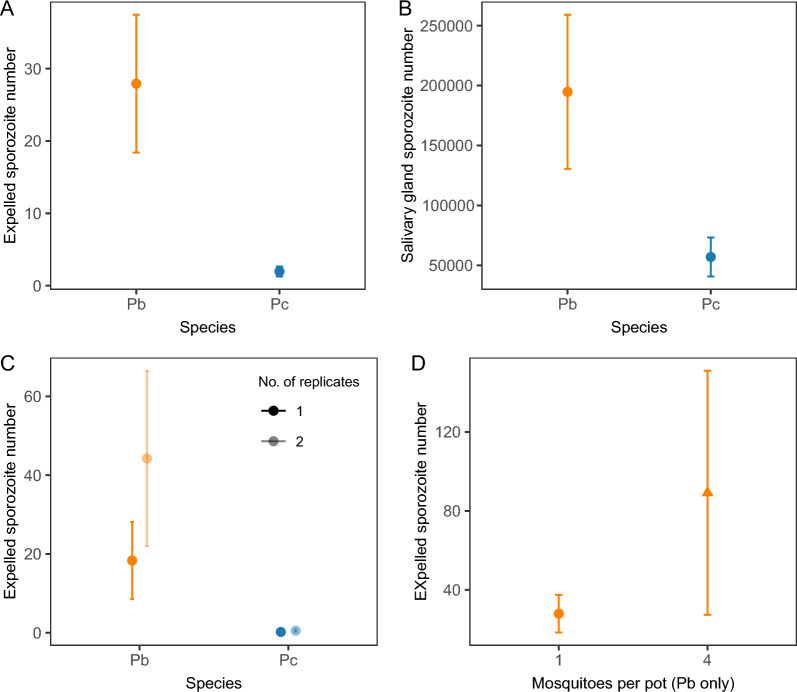
Mean sporozoite densities ± SEM for sporozoite-positive expectorates (**a**, **c**, **d**; per PCR) and salivary glands (**b**; per mosquito) for mosquitoes infected with *P. berghei* (orange) and *P. chabaudi* (blue). Data are presented for single (dark colours) or double (**c**, light colour) qPCR replicates, and for individual mosquitoes (circles) or groups of 4 mosquitoes (triangles, **d**). Pb, *Plasmodium berghei*; Pc, *Plasmodium chabaudi*; qPCR, quantitative-PCR; SEM, standard error of the mean

There was no correlation between the number of sporozoites in the salivary glands of individual mosquitoes and the number of expelled parasites on feeding substrates that tested positive for sporozoites (salivary gland burden:* χ*^2^_(1)_ = 0.01, *P* = 0.92) for either species (salivary gland burden × species interaction:* χ*^2^_(1)_ = 0.84, *P* = 0.36). However, we detected an 11-fold (95% CI: 3.6–34.9) increase in expelled parasites on feeding substrates positive for *P. berghei* compared to those positive for *P. chabaudi* (species:* χ*^2^_(1)_ = 10.7, *P* = 0.001; Fig. 6a). This likely reflects the 3.4-fold (95% CI: 1.2–9.6) higher sporozoite burden in the salivary glands for *P. berghei*-infected mosquitoes compared to *P. chabaudi*-infected mosquitoes (species:* χ*^2^_(1)_ = 4.50, *P* = 0.034; Fig. 6b). The number of sporozoites expelled was 2.9-fold (95% CI: 1.3–6.3) higher on the first versus the second substrate collection day (day:* χ*^2^_(1)_ = 4.8, *P* = 0.028). This may be due to a higher representation of *P. berghei*-infected mosquitoes, with higher sporozoite burdens, in positive substrates on the first (3/4: 75%) versus the second collection day (4/6: 67%) (Table 1).

**Table 1:** Parasite detection rates from sugar-soaked feeding substrates, fed on by an individual mosquito or by groups of mosquitoes

	*P. berghei* (days 23, 25 pIBM)			*P. chabaudi* (days 13, 15 pIBM)		
	Detection rate^a^	Ct^b^	Sporozoites^b^	Det. rate^a^	Ct^b^	Sporozoites^b^
*Individual mosquitoes*						
First collected substrate	0.25 (3/12)	33.6 ± 0.6	47.4 ± 16.5	0.11 (1/9)	37.9	1.5
Second collected substrate	0.33 (4/12)	35.2 ± 0.5	13.3 ± 4.0	0.22 (2/9)	37.5 ± 0.8	2.2 ± 1.1
*Groups of mosquitoes*						
First collected substrate	0.40 (2/5)	33.0 ± 2.3	172 ± 160	n.d	n.d	n.d
Second collected substrate	0.60 (3/5)	34.6 ± 1.2	34.4 ± 21.8	n.d	n.d	n.d

*Ct* Cycle threshold, *n.d* not done,* pIBM *post-infectious blood meal

^a^Proportion of positive substrates (number positive substrates/total tested substrates)

^b^Mean (± standard error of the mean [SEM]) Ct value or mean (± SEM) number of sporozoites per PCR for positive substrates

While 50% (6/12) of *P. berghei*-infected and 33% (3/9) of *P. chabaudi*-infected mosquitoes (confirmed to be positive for salivary gland sporozoites by qPCR) generated at least one positive substrate, the overall proportion of positive feeding substrates from individual mosquitoes was low for both *P. berghei* (29%, 7/24) and *P. chabaudi* (17%, 3/18). A positive substrate on both collection days was found for only one mosquito, infected with *P. berghei*, and the number of positive substrates was similar across days for both species (day:* χ*^2^_(1)_ = 0.63, *P* = 0.43) (Table 1). As expected, the probability of detecting parasites on feeding substrates increased with increasing salivary gland burden (*χ*^2^_(1)_ = 5.92, *P* = 0.015). This trend was observed regardless of species, suggesting that parasites from *P. berghei* and *P. chabaudi*, after adjusting for parasite density, are equally well detected in feeding substrates (salivary gland burden × species interaction:* χ*^2^_(1)_ = 0.30, *P* = 0.58; species:* χ*^2^_(1)_ = 0.004, *P* = 0.95).

To investigate whether the detection rates of parasites on feeding substrates could be improved, we doubled the proportion of the DNA extract quantified by qPCR from 0.4 to 0.9 by running a replicate qPCR reaction. Doubling the volume of sample tested did increase the density of parasites detected across both replicates in positive cotton substrates by twofold (95% CI: 1.0–5.1) (replicate:* χ*^2^_(1)_ = 3.95, *P* = 0.047, Fig. 6c), regardless of species (species × replicate interaction:* χ*^2^_(1)_ = 0.06, *P* = 0.81). However, for each species, the proportion of feeding substrates from which parasite DNA was detected was identical (*P. berghei*: 7/24; *P. chabaudi*: 3/18). Subsequently, we tested if housing mosquitoes in small groups (*n* = 4 mosquitoes) could increase the amount of DNA per feeding substrate (thus improving detection rates), while preserving the possibility to obtain data from replicate groups and track results over time. We used *P. berghei* for this experiment because the higher density of sporozoites in expectorates increases the likelihood that expectorates of multiple mosquitoes will contain detectable sporozoites. As expected, grouping increased the number of expelled sporozoites by threefold (95% CI: 1.1–8.5) (grouping:* χ*^2^_(1)_ = 4.00, *P* = 0.045; Fig. 6d). Fewer sporozoites were expelled on the second collection day, regardless of group size (day × grouping interaction:* χ*^2^_(1)_ = 0.11, *P* = 0.74; day:* χ*^2^_(1)_ = 6.14, *P* = 0.013). However, we did not detect an increase in the rate of detection of *P. berghei* for grouped mosquitoes (grouping:* χ*^2^_(1)_ = 1.31, *P* = 0.25): 50% (5/10) of cotton substrates obtained from groups returned positive expectorate samples, compared to 29.2% (7/24) from individually housed mosquitoes (Table 1).

## Discussion

We tested a non-destructive assay for detecting and quantifying sporozoites from mosquito expectorate. Similar to previous studies [24, 26–28], we demonstrated that DNA from *Plasmodium* sporozoites can be detected from feeding substrates. Moreover, our assay was able to detect rodent *Plasmodium* DNA across a range of concentrations, with no evidence of DNA degradation over 24 h of sample collection, and achieved optimal DNA yield using 8% fructose, which is commonly used to maintain laboratory mosquitoes. However, when testing individual mosquito expectorates (as opposed to reference DNA), the proportion of positive substrates was low.

Our study differs from previous studies in that we investigated individual mosquitoes infected with two commonly used rodent laboratory models, *P. berghei* and *P. chabaudi*. The assay performed similarly for both species, but expectorates from *P. berghei*-infected mosquitoes contained 11-fold more sporozoites than *P. chabaudi-*infected mosquitoes. This is not unexpected considering that *P. berghei* generally reaches higher oocyst and sporozoite densities [31, 43, 44]*,* and there may be malaria species-specific differences in the size of sporozoite inoculum. Indeed, more sporozoites are needed to successfully initiate infections in vertebrate hosts for *P. berghei* compared to *P. yoelii*, suggesting that per-sporozoite infectivity is lower for *P. berghei* [21, 45–47]. We also confirmed previous reports [24, 25] that the rate of detection for sporozoite expulsion (prevalence) increases with higher salivary gland burdens; however, different from [13], we and others [20, 48–50] found no correlation between the number of expelled sporozoites (density) and salivary gland burdens. Therefore, like previous studies using *P. falciparum* [19, 24, 25], our data are consistent with the hypothesis that mosquitoes with higher salivary gland burdens are more likely to expel sporozoites and transmit malaria.

Low detection rates of expelled sporozoites could be due to technical limitations of the assay. The qPCR LOD was four and one genome(s)/PCR for *P. berghei* and *P. chabaudi*, respectively, which is equivalent to 68 *P. berghei* or 14 *P. chabaudi* genomes (i.e. sporozoites) per substrate when taking into account sample processing and DNA recovery. Additionally, proteins present in mosquito saliva can interact with sporozoites [51], potentially reducing the stability of expelled sporozoites, which in turn could raise the detection threshold. Therefore, low densities of expelled sporozoites may have gone undetected. However, in our study, the detection rates did not improve by running multiple technical replicates, nor did they improve for expectorates from groups of four *P. berghei*-infected mosquitoes. Instead, it is more likely that not all substrates contain sporozoites. A similar low prevalence of positive feeding substrates was found by Brugman et al. [28], who reported that 31% (day 21 pIBM) and 55% (day 23 pIBM) of cotton wool DNA extracts were positive for groups of three *P. berghei-*infected mosquitoes; in comparison, 40% (day 23 pIBM) and 60% (day 25 pIBM) of substrates were positive for our mosquito groups. In another study, depending on the mosquito species used, 8–52% of feeding substrates contained DNA of the human malaria parasite *P. falciparum* [24]. Our values of 29% and 17% of total positive substrates collected for individually housed *P. berghei-* and *P. chabaudi*-infected mosquitoes, respectively, fall within this range. In addition, the proportion of individually housed *An. stephensi* mosquitoes generating at least one positive substrate over 24 h (33% for *P. chabaudi* and 50% for *P. berghei*) is within the range observed for *P. falciparum* (35%, Whatman FTA cards [26]; 61%, artificial skin [13]). While higher proportions of mosquitoes with at least one *P. falciparum*-positive cotton substrate (93%) were reported in [24], up to 10 substrates per mosquito were collected, thus increasing the chance of at least one substrate being positive. Taken together, these data indicate that the low detection rate of parasites on feeding substrates may be a common occurrence.

Instead, low detection rates on feeding substrates could be explained by mosquito feeding behaviour, sporozoite biology or a combination of both. While mosquitoes do not expel sporozoites every day [24, 25, 28], female mosquitoes are likely to sugar-feed daily, especially if they do not have access to a blood meal [52]. As the mosquitoes in our study were starved for 24 h in between access to substrates to increase the feeding rate, a lack of sugar-feeding is unlikely to explain the absence of sporozoites on the feeding substrates. It is generally assumed that mosquitoes salivate in a similar way during sugar- and blood-feeding [26, 30], although different enzymes are released from different lobes during the two types of feeding and thus sporozoite expulsion may vary too. Furthermore, sporozoite clumping [53, 54] could increase variation in the expulsion probability and numbers of expelled sporozoites, as observed for *P. yoelii*-infected mosquitoes [21]. *Plasmodium berghei* sporozoites were not detected from 26 days pIBM onwards [28], and we observed lower *P. berghei* sporozoite expulsion on day 25 compared to 23 pIBM, suggesting that sporozoites may degenerate [55] or deplete [56] over time. If so, *Plasmodium* species may vary in terms of sporozoite lifespan in the glands; for example, *P. falciparum* sporozoites have been shown to be expelled for several weeks [24].

How the quantity and quality of sporozoites (both in the salivary glands and expectorate) influences the probability of transmission remains a mystery. While our assay does not test for the infectivity of expelled sporozoites, expelled sporozoites are transcriptionally different to those in the glands [14], and thus may vary in their properties, including infectivity. Therefore, a more appropriate measure of EIP may be the time at which sporozoites are first expelled, rather than when sporozoites appear in the salivary glands, highlighting the need for sensitive non-destructive assays to determine the dynamics of sporozoite expulsion over time. Ideally, the frequency of expulsion should also be accounted for when estimating infectivity throughout a mosquito’s lifespan. While our assay can detect expelled sporozoites from sugar-soaked feeding substrates for two rodent malaria species, like other currently available assays for *P. falciparum* and *P. berghei*, further improvements are needed to track EIP over time in individual mosquitoes. Identifying why detection rates are low remains a key challenge for improving the assay. Our qPCR assay has high sensitivity, so increasing DNA recovery (e.g. a liquid-only feeding system could improve DNA extraction efficiency) is most likely to improve detection rate. Additionally, the frequency of sugar-feeding could be monitored by video or by supplementing sugar with food colouring. Confirming how often mosquitoes feed would allow untouched negative substrates to be excluded and is also key to resolving the likelihood of sporozoite expulsion over time.

## Conclusions

Rodent malaria species are a valuable laboratory tool for comparison between different *Plasmodium* species and for asking broad questions about *Plasmodium* biology. We showed that expelled sporozoites from two different rodent malaria species can be detected from feeding substrates, but further improvement is needed to use this assay for tracking sporozoite expulsion from individual mosquitoes. The low rate of parasite detection in feeding substrates suggests that the appearance and burden of salivary gland sporozoites may not be the most appropriate measure of mosquito infectivity, and that the definition of EIP may require updating. Tracking expelled sporozoites in individual mosquitoes, rather than using salivary gland sporozoite dissections, would be optimal, while also facilitating studies to identify how environment-parasite-vector interactions influence EIP and infectivity to vertebrate hosts over time.

## Data Availability

The datasets supporting the conclusions of this article are available in Edinburgh DataShare repository [https://doi.org/10.7488/ds/7524].
